# AANG Prevents Smad3-dependent Diabetic Nephropathy by Restoring Pancreatic β-Cell Development in db/db Mice

**DOI:** 10.7150/ijbs.72977

**Published:** 2022-08-29

**Authors:** Jeff Yat-Fai Chung, Patrick Ming-Kuen Tang, Max Kam-Kwan Chan, Li Wang, Xiao-Ru Huang, Ka-Fai To, Ronald CW Ma, Hui-Yao Lan

**Affiliations:** 1 Department of Medicine and Therapeutics, Li Ka Shing Institute of Health Sciences, The Chinese University of Hong Kong; 2 Department of Anatomical and Cellular Pathology, State Key Laboratory of Translational Oncology, The Chinese University of Hong Kong; 3 Research Center for Integrated Chinese and Western Medicine, and Department of Cardiology, The Second Affiliated Hospital, Southwest Medical University, Luzhou, Sichuan, China; 4 Guangdong-Hong Kong Joint Laboratory on Immunological and Genetic Kidney Diseases, Guangdong Academy of Medical Sciences, Guangdong Provincial People's Hospital, Guangzhou, China

**Keywords:** Type-2 diabetes, Asiatic Acid, Naringenin, Islet, Nephropathy

## Abstract

Diabetic nephropathy (DN) is a major cause of end-stage kidney disease, where TGF-β1/Smad signaling plays an important role in the disease progression. Our previous studies demonstrated a combination of Traditional Chinese Medicine derived Smad7 agonist Asiatic Acid (AA) and Smad3 inhibitor Naringenin (NG), AANG, effectively suppressed the progression of renal fibrosis *in vivo*. However, its implication in type-2 diabetic nephropathy (T2DN) is still unexplored. Here, we detected progressive activation of Smad3 but reduction of Smad7 in db/db mice during T2DN development. Therefore, we optimized the dosage and the combination ratio of AANG to achieve a better rebalancing Smad3/Smad7 signaling for treatment of T2DN. Unexpectedly, preventive treatment with combined AANG from week 4 before the development of diabetes and T2DN effectively protected against the onset of T2DN. In contract, these inhibitory effects were lost when db/db mice received the late AANG treatment from 12-24 weeks. Surprisingly, preventive treatment with AANG ameliorated not only T2DN but also the primary disease type-2 diabetes (T2D) with relative normal levels of fasting blood glucose and HbA1c, and largely improving metabolic abnormalities especially on insulin insensitivity and glucose tolerance in db/db mice. Mechanistically, AANG effectively prevented both Smad3-mediated renal fibrosis and NF-κB-driven renal inflammation in the diabetic kidney *in vivo* and advanced glycation end-products (AGE) stimulated tubular epithelial mTEC cells *in vitro*. More importantly, we uncovered that preventive treatment with AANG effectively protected against diabetic-associated islet injury via restoring the β cell development in db/db mice. Taken together, we discovered that the early treatment with combined AANG can effectively protect against the development of T2D and T2DN via mechanism associated with protection against Smad3-depenedent islet injury.

## Introduction

Diabetes mellitus affects 346 million people worldwide and 582.5 thousand Hong Kong population with 10-20% mortality due to kidney failure (International Diabetes Federation 2015, Centers for Disease Control and Prevention 2011). Its onset and progression are multifactorial but largely due to the development of systemic insulin resistance and insufficient insulin secretion 1-3. Although a significant progress has been made in our understanding of the pathogenesis of type-2 diabetic nephropathy (T2DN), its treatment remains ineffective. Therefore, it is an urgent need for searching and developing strategies that can prevent and effectively attenuate its disease progression.

In patients with T2DN, TGF-β/Smad3 signaling is highly activated, which is companied with the loss of its inhibitor Smad7, resulting in significant glomerular and tubulointerstitial fibrosis 4. Thus, the Smad signaling pathway is dysregulated and imbalanced within the diabetic kidney. It has been reported that high glucose, AGE and Ang II can activate Smad2 and Smad3 to induce collagen matrix synthesis by mesangial cells, tubular epithelial cells, and vascular smooth muscle cells *in vitro* 4-6. It is now well established that Smads act as signal integrators and interact with other signaling pathways to mediate DN 3, 7. In the context of renal fibrosis, Smad3, a key downstream of mediator, is pathogenic, but Smad7, a negative regulator of Smad3, is protective 4-6. In addition, Smad7 is also an inhibitor of NF-κB signaling by inducing IκBα and can also inhibit NFκB-dependent renal inflammation including diabetic and hypertensive nephropathy 7, 8. Therefore, deletion of Smad3 or overexpression of Smad7 protects kidney from Smad3-mediated renal fibrosis and NFκB-driven renal inflammation under diabetic and hypertensive conditions 3, 7-11. Furthermore, most recent studies also revealed that Smad3 can transcriptionally suppress islet β cell development and insulin production as Smad3 can directly target Pdx-1, Ins1, Ins2, Nkx6.1, Glp1-R and NeuroD1 12, 13, implying that Smad3 transcriptionally regulates islet β cell proliferation and function to influence the progression and regression of T2D 14. This is confirmed by the finding that Smad3 deficiency in mice largely improves insulin resistance and obesity induced by a high-fat diet 15. Our recent study also found that db/db mice lacking Smad3 are protected from the development of diabetic phenotype via protecting islet β cells from the diabetic injury 16. Thus, islet β cells lacking Smad3 provide a better therapy for diabetes and diabetic kidney disease 17. Taken together, all these studies reveal that targeting Smad3 may not only inhibit T2DN but also improves the primary T2D.

Traditional Chinese medicine (TCM) has been widely used for the treatment of diabetes and its complications in China. We have recently reported that Asiatic acid (AA) is a Smad7 agonist and Naringenin (NG) is a Smad3 inhibitor 9. The combination treatment of two active compounds isolated from TCM significantly inhibited chronic kidney disease associated renal fibrosis by rebalancing TGF-β/Smad signaling on a well-characterized mouse model of unilateral ureteral obstruction (UUO) 9. In the present study, we further evaluated the therapeutic potential of the combined treatment with AA and NG on T2DN in vivo and *in vitro*. Here, we successfully developed an AANG formula that can effectively rebalance renal TGF-β/Smad signaling and thus prevents the development of T2DN. Unexpectedly, we discovered that AANG protected db/db mice from diabetic islet loss via promoting β cell development, which markedly decelerated the development of type-2 diabetes (T2D), thereby preventing T2DN. Thus, AANG may represent as a novel, safe and effective strategy for T2D and T2DN prevention by effectively rebalancing TGF-β/Smad signaling.

## Methods

### Animal model and AANG treatments

AA is a HLPC-purified product (95%) purchased from Guangxi Changzhou Natural Pharmaceutical Co. Ltd (Nanning, Guangxi, China). NG is obtained from Shanxi Huike Botanical Development Co. Ltd (Xian, Shanxi, China) with the HLPC-purity of 98%. The db/db mice were treated with oral AA (30 or 300 mg/kg/day), NG (300 or 3000 mg/kg/day), or combination of AA and NG (AANG in 1:10 ratio) by daily food-intake from the pre-diabetic age of 4 to 24 weeks (preventive treatment) or in the established T2D and T2DN mice from the age of 12 to 24 weeks (intervention treatment). Untreated db/db and db/m mice served as control groups. Finally, all mice were sacrificed via injection of ketamine/xylene. All studies were approval by the Animal Experimentation Ethics Committee, the Chinese University of Hong Kong and the experimental methods were carried out in accordance with the approved guidelines.

### Fasting Blood Glucose, HbA1c, Glucose and Insulin Tolerance Tests

Blood glucose levels were measured by Accu-Chek glucose meter (Roche Diagnostics) after the mouse fasting for 6 hours as our previous study 10. HbA1c were measured by mouse HbA1c ELISA kit following the manufacturer manual (Cat.: 80310, Crytal Chem).

The glucose tolerance test (IPGTT) was performed as previously described 5, 6, mice were fasted 6 hours and given i.p. injection of glucose (2 mg/g body weight). Blood glucoses were determined at 5-, 15-, 30-, 60- and 120-minutes post injection. For insulin tolerance tests (IPITT), mice were fasted for 6 hours and given i.p. injection of insulin (1 U/kg). Blood glucoses were determined at 5-, 15-, 30-, 60- and 120-minutes post injection as previously described 16,17.

### Histology and Immunohistochemistry

Kidneys sections were fixed in 4% paraformaldehyde, stained with the Periodic Acid-Schiff (PAS) method. Immunohistochemistry was carried out by using a microwave-based antigen retrieval technique. The primary antibodies used in the current as previous described 11-14. The nuclei were counterstained with haematoxylin. The positive cells were counted under the power field of microscope in 10 random areas of kidney tissues with expected as cells/mm, and the percentage of positive staining areas was quantified using the Image-Pro Plus software (Media Cybernetics, Bethesda, MD) in 10 consecutive fields.

### Cell culture

The mouse tubular epithelial cells (mTEC) were cultured in DMEM/F12 (Gibco, CA), supplemented with 10% FBS (Gibco, CA) and 1% antibiotic/antimycotic solution (Life Technologies, USA). The mouse mesangial cells (mMES) (MES13 ATCC, Manassas, VA, USA) was maintained in a 3:1 mixture of DMEM and Ham's F-12 medium containing 5% FBS, and 1% antibiotic/antimycotic solution (Life Technologies, Grand Island, NY, USA)^10^, ^12^, ^15^. mTECs were seeded and treated with combination of AANG (20 µM AA+200 µM NG) in a 6-well plate for 6-hours, then stimulated with advanced glycation end-products (AGE) for 24-hours. Subsequently, stimulated mTECs were employed for western blot analysis.

### MTT assay

The MTT assay was used to determine the cytotoxicity of AANG on both mTES and mMES cells *in vitro* following our previous procedure 16. In brief, cells (1x10^4^/well) were seeded on a 96-well plate and serial concentrations of AA, NG, or their combination with indicated concentration were added on the next day. After 24-hours of treatment, 30μl methyl-thiazoldiphenyl tetrazolium (MTT) (5 mg/ml) were added to each well and incubated for 2 h at 37°C. The MTT solution was then replaced by 100 μl dimethyl sulfoxide in each well and measured with a microtiter-plate reader (BioTek) at 540 nm and all data were calculated as percentage against the control.

### Western blot Analysis

Protein from renal cortex and cultured renal cell lines was extracted using the radio immunoprecipitation assay (RIPA) lysis buffer. Western blot analysis was performed as pervious described 12. Antibodies involved such as collagen-I (Col-I), α-smooth muscle actin (α-SMA), phospho-NF-kB/p65, phospho-Smad3, Smad3, Smad7 and β-actin were described as previous 14, 15. Then, IRDye800-conjugated secondary antibodies (Rockland Immunochemical, Gilbertsville, PA) were used as secondary antibodies. Signals were detected using the LiCor/Odyssey infrared image system (LI-COR Biosciences, Lincoln, NE), followed by quantitative analysis using the Image J program.

### RNA Extraction, Quantitative Real-Time PCR

Total RNA was extracted from the renal cortical tissues and cultured renal cell lines. Real-time PCR was carried out with machine (Option 2, Bio-Rad, Hercules, CA, USA) by using IQ SYBR Green Supermix reagent (Bio-Rad) 16, 17. The Sequence of RNA Primers used such as Smad3, Smad7, collagen-I, α-SMA, MCP-1, NF-kB, ERBB4-IR, LRNA9884, and GAPDH were described previously 5, 7, 12, 18. The house keeping genes β-actin was used as internal controls. The ratio of specific mRNA to β-actin mRNA was calculated using the 2^-ΔCt^ method and is expressed as the mean ± S.E.M.

### Renal Function Measurement

24-hours urinary samples of mice were collected in metabolic cages every 4 weeks from the age of 4 weeks to 24 weeks (for prevention study) and 12 weeks to 24 weeks (for intervention study). Urinary microalbumin was measured by competitive ELISA according to the manufacturer's instructions (Exocell, PA). Levels of blood and serum creatinine were determined accordingly with enzymatic method (Stanbio Laboratories, TX, USA). Urinary albumin excretion was expressed as total urinary albumin/creatinine (μg/mg) as previously study. All measurements were performed as previously described 10.

### Measurement of Serum Lactate dehydrogenase (LDH), Alanine aminotransferase (ALT) and Aspartate aminotransferase (AST) levels

Commercial kits Stanbio-ALT/SGPT Liqui-UV® Test and AST/SGOT Liqui-UV® Test from Stanbio Laboratory were used. QuantiChrom™ Lactate Dehydrogenase Kit (DLDH-100) used for LDH detection was purchased from BioAssay as previous studies 14, 19.

### Immunofluorescence Staining

Immunofluorescence staining was performed with 5 μm PLP-fixed frozen sections. Primary antibodies were incubated overnight, then followed by RHO-conjugated anti-mouse and fluorescein isothiocyanate (FITC)-conjugated anti-rabbit secondary antibodies, respectively 15, 20. All slides were mounted with DAPI-containing mounting medium and then analysed with a fluorescence microscope (Leica Microsystems, Wetzlar, Germany).

### Statistical Analysis

All the data are expressed as mean ± SEM. Statistical analyses were performed with one-way analysis of variance (ANOVA), followed by Newman-Keuls multiple comparison from GraphPad Prism 5.0 (GraphPad Software, San Diego, CA). In addition, a repeated analysis ANOVA analysis was used for albumin excretion, body weight, fasting blood glucose analysis.

## Results

### AANG formula for rebalancing Smad3/Smad7 equilibrium in db/db mice

**Figure 1:**
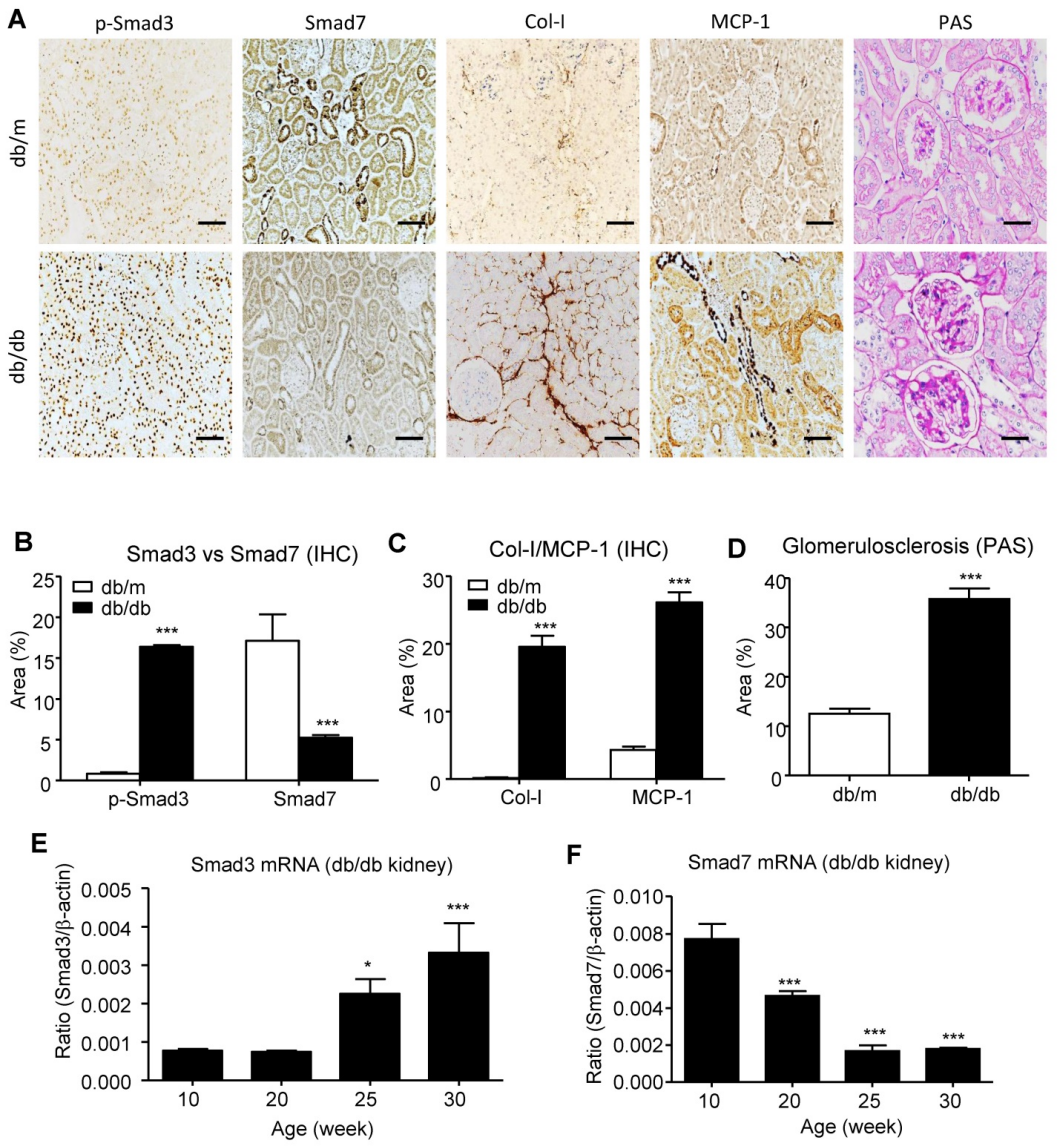
** Imbalance of renal Smad3/Smad7 equilibrium in T2DN. (A-D)** Changes in renal Smad3/Smad7 equilibrium in the diabetic kidney of db/db mice with overreactive Smad3 but loss of Smad7 is highly associated with the T2DN development by increasing collagen I and MCP-1 expression in 24-week-old db/db mice. **(E, F)** Immunohistochemistry and real-time PCR analysis show a progressive increase in Smad3 activation but decrease in Smad7 expression in the kidney of db/db mice. Data represent mean ± s.d. from groups of 5 mice. (B-D) ***p<0.001 vs 24-week-old db/m mice. (E, F) *p<0.05, **p<0.01, ***p<0.001 vs 10-week-old db/db mice. Scale bar, 50 μm.

**Figure 2:**
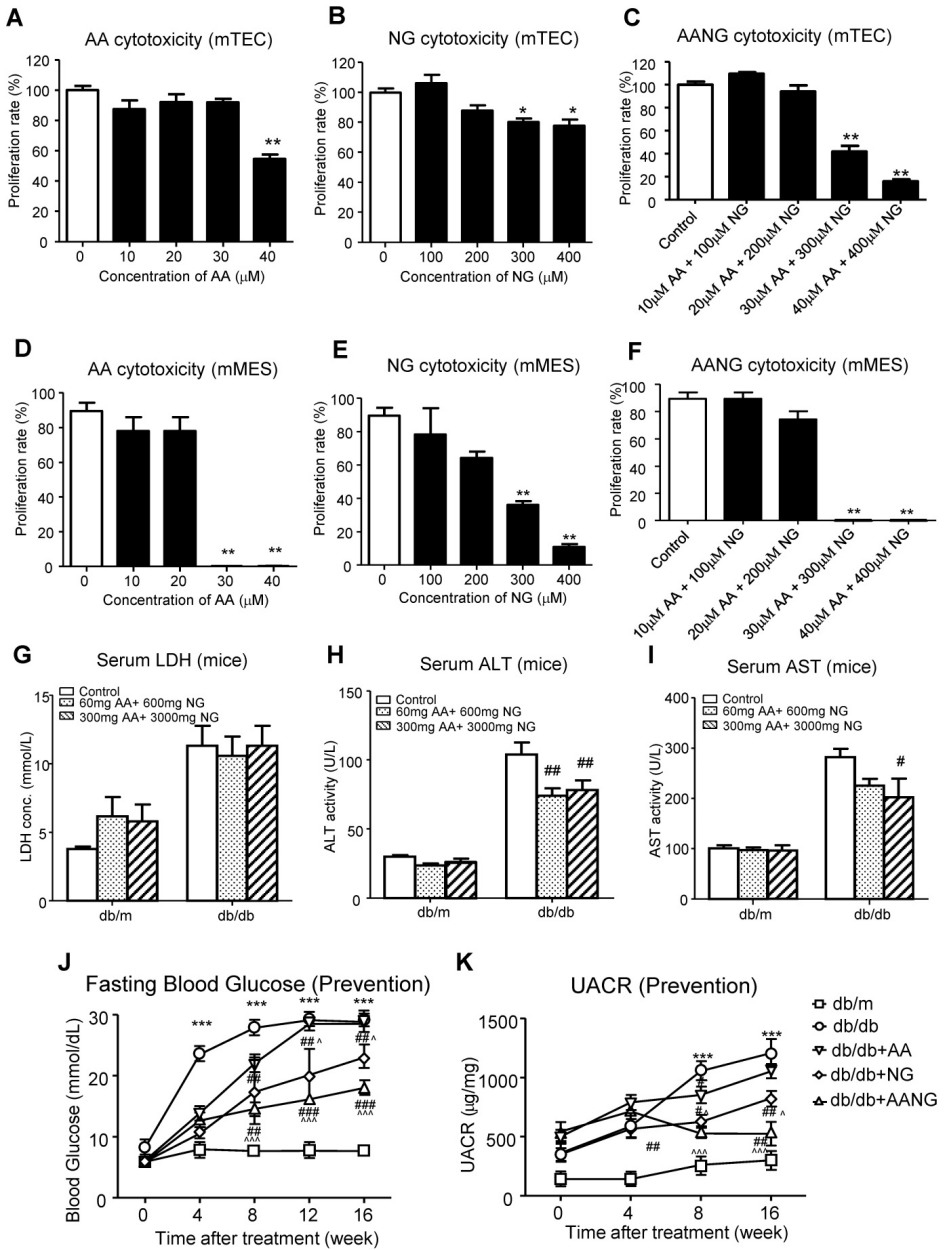
** Identification of optimal AANG formula for treatment of db/db mice. (A-F)** *in vitro* cytotoxicity assay by MMT. Cultured murine renal tubular epithelial cells mTEC **(A-C)** and mesangial cells mMES **(D-F)** were treated with AA, NG, or the combination with indicated dose, the cell proliferation rates were detected by MTT assay at 24h *in vitro*. **(G-K)** The db/m or db/db mice received the combination of AA and NG with indicated doses daily from rodent food for 20 weeks since 4-week-old for measuring the serum levels of LDH, ALT, AST, fasting blood glucose and UACR. Data represented mean ± s.d. from groups of 5 mice. (A-F) *p<0.05, **p<0.01 vs control; (G-K) ***p<0.001 vs db/m; #p<0.05, ##<0.01, ###p<0.001 vs db/db; ^p<0.05, ^^^p<0.001 vs db/db+AA.

We have previously demonstrated that the combination treatment of AA and NG can effectively rebalance the Smad3 and Smad7 signaling in the acute kidney injury model 9 and tumor microenvironment 14. Here, we also found the shift of Smad3/Smad7 equilibrium in renal microenvironment of db/db mice was highly associated with their T2DN development, where renal Smad3 was highly activated, but Smad7 was suppressed at both protein and mRNA levels (**Figure 1**). We first optimized AANG, the combined formula of AA and NG, specifically for targeting T2DN by conducting cytotoxicity screening assays *in vitro* and *in vivo*. The optimal ratio of AA and NG individually on cultured mTEC and mMES cells was determined by MTT assay. We identified that 20µM of AA and 200µM of NG were safe for either individual or combined usages, showing by their insignificant cytotoxicity on the epithelial mTEC and mesangial mMES cells at 24h *in vitro* (**Figure 2A-F**). Therefore, we combined AA and NG in the ratio of 1:10 and tested the safety and therapeutic effect *in vivo*. The 10-weeks-old db/m and db/db mice were treated with AANG at the indicated dosages. Interestingly, compared to control db/db mice, high dose of AANG (300mg AA+3000mg NG) showed protective effects on diabetic liver injury according to their significant reduction of serum ALT and AST levels without alteration of serum LDH (**Figure 2G-I**). Importantly, this dosage of AANG also showed to effectively protect db/db mice from the development of diabetes and T2DN by significantly lowering fasting blood glucose and microalbuminuria when compared to the untreated control as well as their monotherapy *in vivo* (**Figure 2J, K**). Thus, we identified 300mgAA+3000mgNG as an optimal AANG formula for db/db mice.

### AANG effectively prevents onset of T2DN in db/db mice

**Figure 3:**
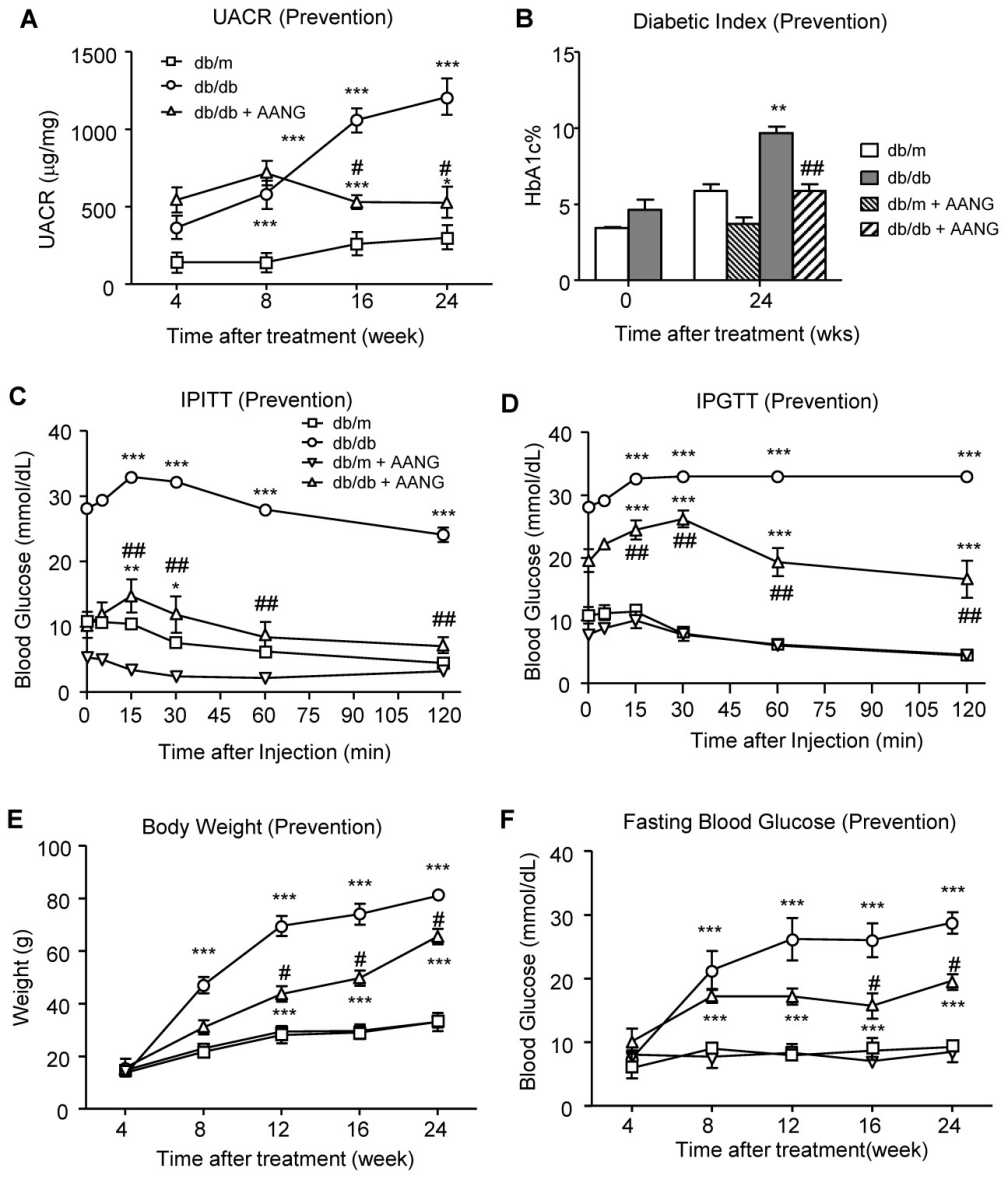
** Preventive treatment with AANG effectively ameliorates the T2D-associated kidney injury and metabolic abnormalities in db/db mice. (A)** The 20-week-preventive treatment with an optimal dose of AANG (300mg/kg/day AA and 3000mg/kg/day NG) from week 4 to week 24) effectively improves the renal dysfunction of db/db mice by significantly reducing UACR. **(B)** Unexpectedly, AANG preventive treatment also markedly normalizes the diabetic index HbA1c level of db/db mice to be insignificant with db/m mice. In addition, AANG preventive treatment not only improves the **(C)** insulin sensitivity (IPITT) and **(D)** glucose tolerance (IPGTT), but also reduces the **(E)** body weight and **(F)** fasting blood glucose levels in db/db mice. Data represented mean ± s.d. from groups of 6 mice. **p<0.01, ***p < 0.001 vs control db/m mice; #p<0.05, ##p<0.01 vs control db/db mice.

After determining the safe and effective dosage of AANG combination, we first examined whether AANG has preventive effect on T2D and T2DN. Since TCM can effectively protect against a number of inflammatory diseases 21, we first examined the preventive effect of AANG on T2DN by treating pre-diabetic db/db mice with AANG from 4 weeks to 24 weeks. Encouragingly, according to the urine albumin-to-creatinine ratio (UACR) in **Figure 3A**, preventive treatment with the combination of AA (300mg/kg/day) and NG (3000mg/kg/day) significantly protected db/db mice from the development of microalbuminuria. Surprisingly, we also detected that the provoked diabetic index HbA1c level in db/db mice was also markedly reduced in the preventive -treated db/db mice to the normal level as db/m mice (**Figure 3B**). In addition, the metabolic abnormalities of db/db mice were significantly ameliorated by AANG preventive treatment (db/db+AANG) by dramatically improving the insulin sensitivity and glucose metabolism (**Figure 3C, D**) and reduced glucose tolerance, body weight and fasting blood glucose levels (**Figure 3E, F**). These findings unexpectedly uncovered a promising preventive effect of AANG on T2DN as well as its primary disease T2D.

In this study, we also examined the therapeutic potential of AANG on established T2DN by treating db/db mice with AANG from week 12 to week 24. According to **Supplementary Figure S1A**, the 12-weeks-treatment with the combination of AA (300mg/kg/day) and NG (3000mg/kg/day) from the age of week 12 was not able to improve the diabetic renal injury compared to their control group (db/db). No significant changes were also detected in the diabetic index HbA1c, insulin sensitivity (IPITT), body weight, and fasting blood glucose level in the AANG-treated db/db mice compared to their untreated db/db group (**Supplementary Figure S1B-F**), although a significant improvement in glucose tolerance (IPGTT) was achieved in AANG-treated db/db mice (**Supplementary Figure S1D**). By comparing with the clinical outcomes achieved in the preventive treatment, results from the intervention therapy suggested that AANG is more suitable for the early prevention rather than the late treatment for the established T2D and T2DN. Thus, the working mechanism of AANG for T2DN prevention was intensively elucidated in this study.

### AANG prevents T2DN by inhibiting Smad3-mediated renal fibrosis and NF-κB -driven renal inflammation

**Figure 4:**
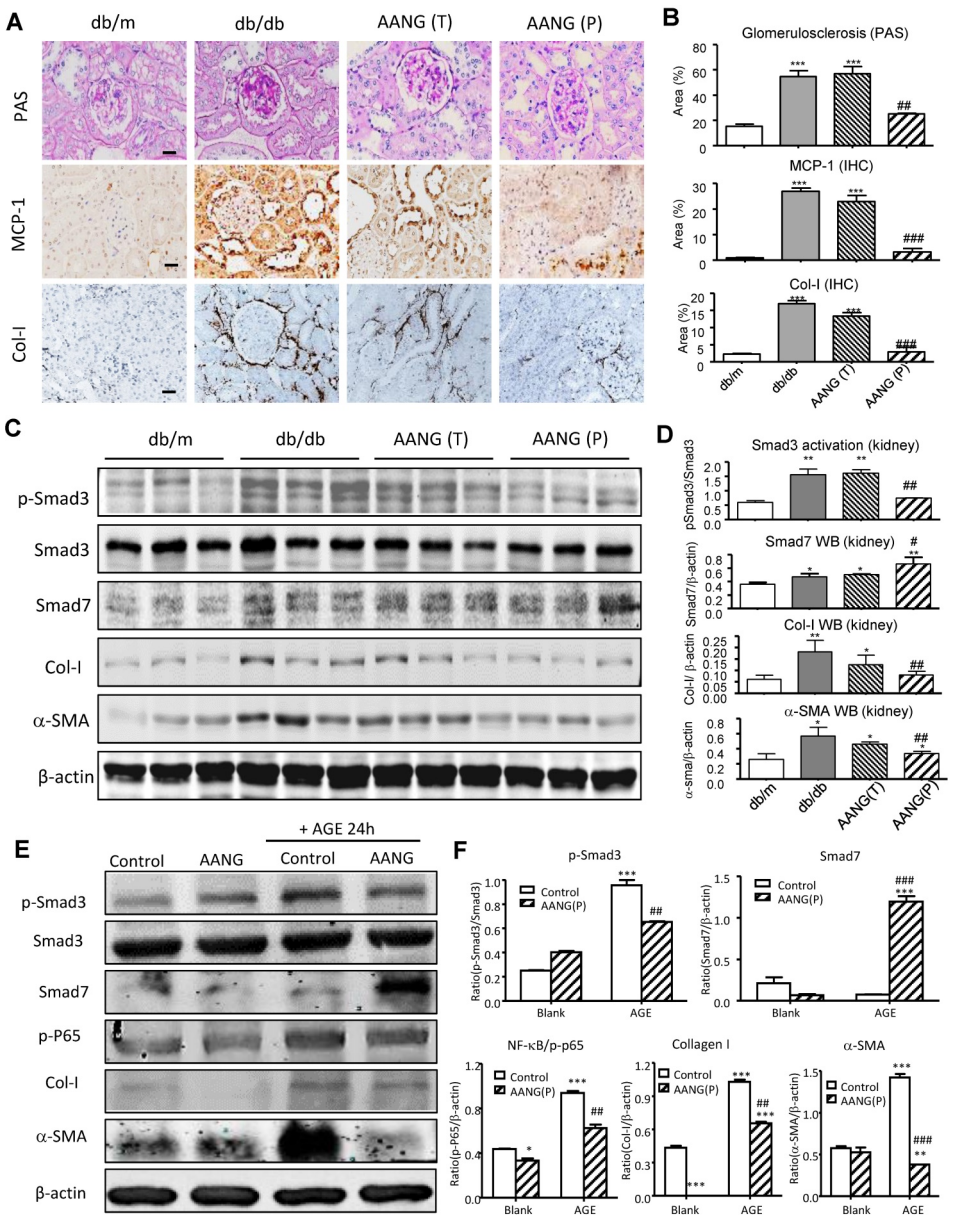
** Preventive but not intervention treatment with AANG effectively blocks T2DN development in db/db mice. (A, B)** Immunostaining shows that renal inflammation and fibrosis in the diabetic kidney of db/db mice are markedly suppressed by AANG preventive treatment (P) but not by intervention therapy (T).** (C-F)** Western blot analysis shows the Smad3 activation (p-Smad3) and increment of fibrotic genes collagen I and α-SMA in the diabetic kidney of db/db mice are markedly suppressed by AANG preventive treatment (P) but not by intervention therapy (T); where Smad7 expression is synergistically increased by AANG preventive treatment in the **(C, D)** kidney of db/db mice at 24 weeks. **(E, F)** Effects of AANG on AGE-induced activation of Smad3 and NF-κB signaling expression collagen I and α-SMA by mTEC at 24h. Data represented mean ± s.d. from groups of 6 mice or independent experiments. (B, D) *p<0.05, **p < 0.01 vs db/m mice; #p<0.05, ##p<0.01 vs control db/db mice. (F) *p<0.05, ***p < 0.001 vs blank control; ##p<0.01, ###p<0.001 vs AGE-treated control. Scale bar, 50 μm.

**Figure 5:**
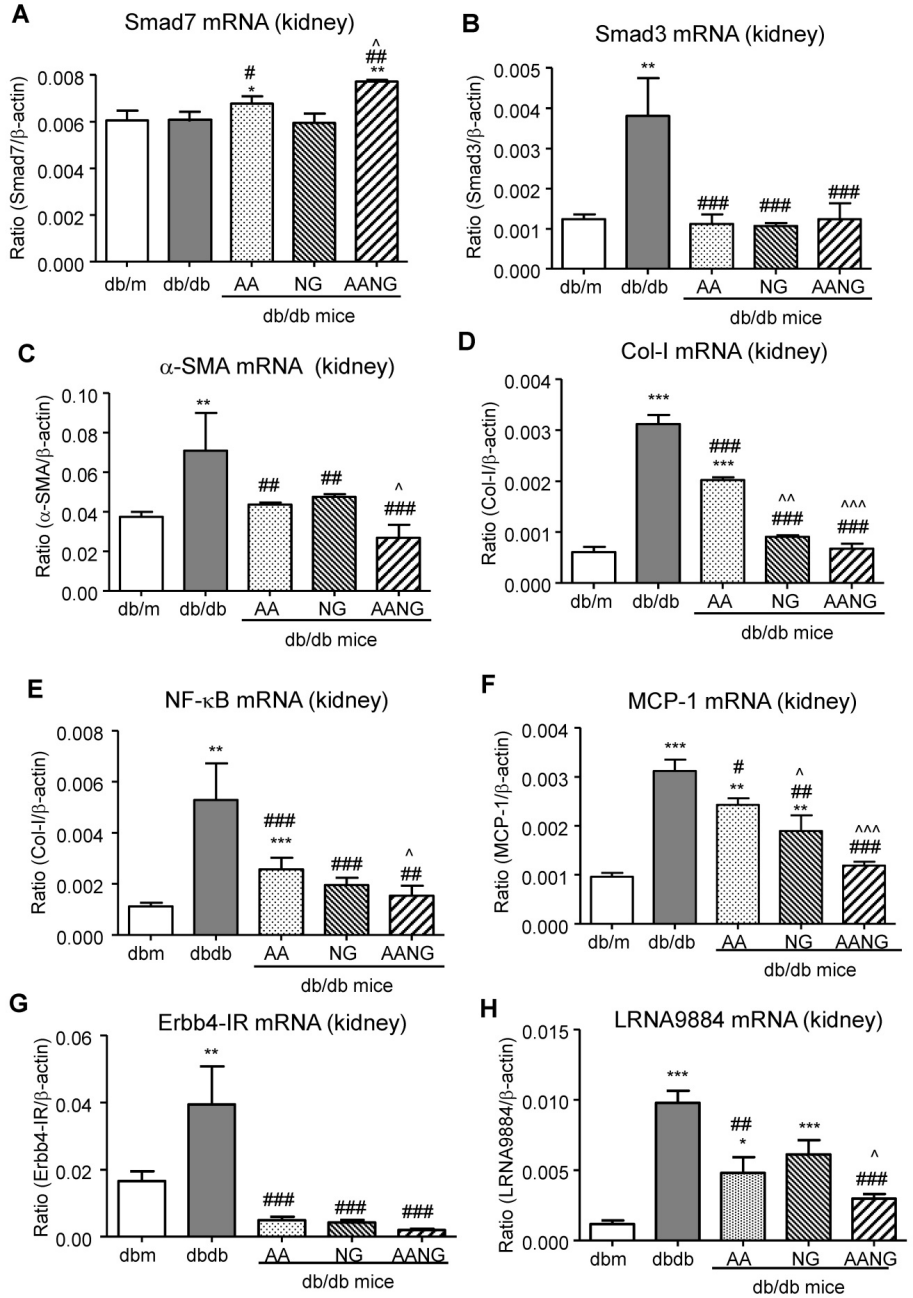
** AANG shows a better preventive effect on rebalancing Smad3/7 and inhibiting NF-κB signaling as well as lncRNA Erbb4-IR and LRNA9884 expression in the diabetic kidney of db/db mice at 24 weeks. (A-D)** Real-time PCR analysis shows that compared to AA or AG monotherapy, AANG effectively increases renal expression of anti-inflammatory Smad7 mRNA while inhibiting Smad3, which is associated with inhibition of renal fibrosis including α-SMA and collagen I mRNA expression. **(E, F)** Real-time PCR detects that preventive treatment with AANG produces a better inhibitory effect on NF-κB activation and MCP-1 mRNA expression. **(G, H)** Effects of AANG preventive treatment on Smad3-dependent lncRNAs Erbb4-IR and LRNA9884 in the diabetic kidney of db/db mice in 24 weeks. Data represented mean ± s.d. from groups of 6 mice. *p<0.05, **p < 0.01, ***p<0.001 vs db/m mice; #p<0.05, ##p<0.01. ###p<0.001 vs control db/db mice; ^p<0.05, ^^p < 0.01, ^^^p<0.001 vs AA-treated db/db mice.

Increasing evidence demonstrated that TGF-β1/Smad3 signaling is hyperactivated in diabetic kidney and critical for T2DN development 12, 15, 22. Our previous studies demonstrated the combined treatment of AA and NG effectively inhibited Smad3-mediated renal fibrosis in the UUO-injured mouse model 9. Here, we observed that the preventive but not intervention therapy with AANG produced a profound effect on T2DN in mice by protecting the kidney from diabetic pathology and renal expression of MCP-1 and collagen I deposition (**Figure 4A and B**). We thus examined the mechanisms of preventive versus intervention therapy with AANG on Smad3-mediated renal fibrosis. As shown in **Figure 4C and D**, western blot analysis detected that Smad3 was highly activated in the kidney of db/db mice compared to db/m mice, which was markedly suppressed by the AANG preventive-treatment (P) but not by the intervention treatment (T). In contrast, AANG preventive treatment significantly enhanced renal Smad7 expression **(Figure 4C and D),** resulting in inhibition of renal fibrosis by reducing collagen I and α-SMA expression in the diabetic kidney of db/db mice **(Figure 4A-D)**. These observations were also confirmed by real-time PCR at the mRNA levels. Interestingly, preventive treatment with AA, NG or AANG produced a differential effect on upregulation of renal Smad7 while inhibiting Smad3 mRNA expression (**Figure 5 A, B),** thereby blocking α-SMA and collagen I mRNA expression in db/db mice (**Figure 5 C, D).**

It is well established that inflammatory NF-kB signaling plays a promoting role in T2DN, which can be suppressed by Smad7 overexpression *in vivo* 23, 24. Thus, we also investigated the potential inhibitory effect of AANG treatment on NF-κB-driven renal inflammation by real-time PCR. We found that AA (but not NG) significantly increased Smad7 transcription while inhibiting NF-κB and MCP-1 mRNA expression in the kidney of db/db mice, which was further enhanced in db/db mice treated with AANG (**Figure 5A, E, and F**).

To further examine the mechanisms by which AANG inhibits T2DN, we performed an *in vitro* study in mTEC cells. In line with the findings from *in vivo* study (**Figure 4 C, D**), we demonstrated that AANG pre-treatment markedly upregulated Smad7 while significantly inhibiting AGE-induced Smad3 as well as NF-κB activation, thereby inhibiting collagen I and α-SMA expression in the mTEC *in vitro* (**Figure 4E, F**). Taken together, these findings clearly demonstrated that AANG preventive treatment effectively inhibits Smad3-mediated renal fibrosis and NF-κB -driven renal inflammation, thereby protecting the kidney from the development of T2DN in db/db mice.

We have also previously found that Smad3-mediated T2DN via a number of long noncoding RNAs (lncRNAs) including fibrotic Erbb4-IR 12 and inflammatory LRNA9884 5, 15. We thus examined whether preventive treatment with AANG inhibited expression of Erbb4-IR and LRNA9884 in the kidney of db/db mice. As shown in **Figure 5 (G, H),** preventive treatment with AA, NG, and AANG significantly blocked Erbb4-IR and LRNA9884 expression in the diabetic kidney. Interestingly, compared to monotherapy with AA or NG, combinational therapy with AANG produced a synergistic effect on the LRNA9884 suppression in db/db mice (**Figure 5F**).

### AANG prevents T2D and T2DN by promoting islet β cell development

**Figure 6:**
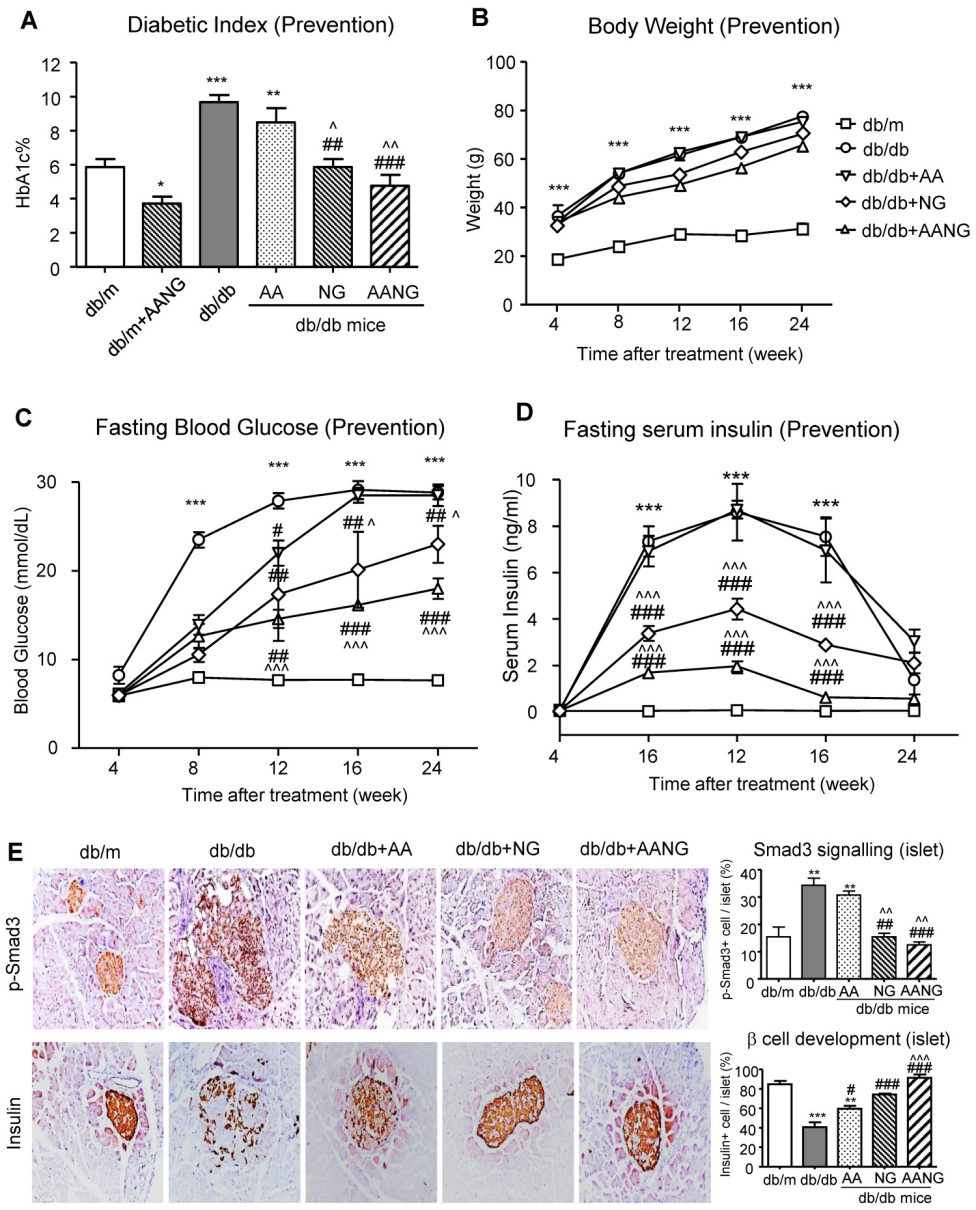
** Preventive treatment with AANG effectively inhibits diabetes in db/db mice by promoting islet β cell development. (A-D)** Preventive treatment with AANG results in normal levels of fasting blood glucose, HbA1c, and fasting serum insulin in db/db mice without affecting body weight. Note that monotherapy with NG but not AA also produces a better hyperglycaemia control, which is further enhanced by AANG treatment **(E)** Immunohistochemistry shows that compared to AA or NG monotherapy, preventive treatment with AANG markedly blocks Smad3 signaling while increasing insulin production in the diabetic islets at 24-week db/db mice. Data represented mean ± s.d. from groups of 6 mice. *p<0.05, **p < 0.01, ***p<0.001 vs db/m mice; #p<0.05, ##p<0.01. ###p<0.001 vs control db/db mice; ^p<0.05, ^^p < 0.01 vs AA-treated db/db mice. Scale bar, 50 μm.

Previous studies have shown that Smad3 can target islet β cells to cause T2D 12-17. In the present study, we also revealed that preventive treatment with AANG suppressed T2D and T2DN in db/db mice (**Figures 3 and 4**). We therefore further elucidated the underlying mechanism of AANG in preventing the development of T2D and T2DN. As shown in **Figure 6 (A-D)**, treatment with NG but not AA prevented the development of T2D in db/db mice with relative normal levels of HbA1c, fasting blood glucose, and serum insulin levels, which became more effective in db/db mice received AANG treatment. These observations are in line with the anti-diabetic effects found in the Smad3-KO db/db mice 25, 26, revealing Smad3 as a key transcriptional factor and therapeutic target for T2D and T2DN. This was further demonstrated by the DIAMANTE T2D GWAS that the abundancy of active transcription start site was enriched on Smad3 gene, especially in pancreatic islets of type-2 diabetes patients compared to other tissues such as liver, adipose tissue, and skeletal muscle (**Supplementary Figure S2**). It is known that db/db mice or islet cells lacking Smad3 are protected from T2D 16,17. We thus examined if the preventive effect of AANG on T2D and T2DN is associated with the inhibition of islet β cell injury in Smad3 KO-db/db mice. Two-color immunofluorescence showed that a significant increase in phosphorated Smad3 in islet cells of Smad3 WT-db/db mice was associated with a loss of the insulin-producing islet β cell, which was reversed in Smad3KO-db/db mice with a marked increase in insulin-producing β cells (**Supplementary Figure S3**). Like the Smad3 KO-db/db mice, we uncovered that compared to the AA or NG monotherapy, preventive treatment with AANG also markedly blocked Smad3 activation in the diabetic pancreas, resulting in the protection of islet from the degenerative changes and the restoration of islet β cell mass and insulin production in db/db mice (**Figure 6E**). Taken together, we discovered that the protection against Smad3-mediated islet deterioration may be a novel mechanism through which AANG treatment prevents T2D and T2DN.

## Discussion

Diabetic nephropathy (DN) is a severe complication of T2D, and one of the major causes of ESRD, but effective therapies are still limited due to its unclear pathogenic mechanism. It is well documented that both NF-kB and Smad3 signaling pathways were elevated in T2D patients and experimental disease models 7, 27, 28. Recently, we developed a TCM-derived natural compound formula AANG effectively inhibited acute kidney injury as well as cancer progression by rebalancing the Smad3/7 equilibrium in the inflammatory microenvironment 9, 14. Here, we examined the therapeutic potential of AANG on T2D and T2DN and identified its safe dosage window and optimal combination ratio that effectively protected db/db mice against T2D and T2DN development by blocking both NF-kB-mediated renal inflammation and Smad3-mediated renal fibrosis *in vitro* and *in vivo*. Surprisingly, we further discovered that AANG could directly inhibit the progression of T2D by restoring β cell development in db/db mice via inhibiting the Smad3-dependent islet injury. Thus, AANG may represent as a novel and effective therapeutic for T2D and T2DN prevention.

The therapeutic effects of TCM have been well-documented for T2D and its complications 29, 30. In the present study, we identified 300mg/kg/day AA and 3000mg/kg/day NG as the safe and effective dosage for treatment of T2D and T2DN in db/db mice and found that the combination of AA and GN at the ratio of 1:10 was an optimal dosage to produce a profound preventive effect on the development of T2D and T2DN in db/db mice if treatment was beginning early from the pre-diabetic stage at the age of 4 weeks. However, these protective effects were lost if the treatment was conducted late from the age of 12 weeks when the T2D and T2DN is established in db/db mice. Findings from this study implied that AANG may represent the active compound in TCM for preventing T2D and diabetic complications including diabetic nephropathy. These findings are significant clinically and suggested that treatment for T2D and diabetic complications with TCM should start as early as possible, with much more effective at the early or prediabetic stage. The failure to achieve the therapeutic effect on T2DN-established db/db mice also suggested that much more intensive diabetic controls are needed in patients with T2DN.

Mechanically, we uncovered that the combinational treatment with AA and NG produced a better effect on rebalancing Smad3/Smad7 signaling in the diabetic kidney, thereby resulting in more effectively inhibiting Smad3-mediated renal fibrosis and NF-κB-driven renal inflammation. Indeed, the Smad signaling network, especially Smad3, has been shown to play a pathogenic role in renal fibrosis including T2DN 14, 16, 31, 32. Furthermore, Smad3 is also essential for regulating islet cell proliferation and development 33, 34. Consistent with these notions, in this study, we detected a significant association between Smad3 hyperactivation with pancreatic islets of T2D patients by GWAS as well as islet degeneration in db/db mice. Importantly, we also noticed that preventive treatment with AANG was able to protect the islet β cells from diabetic injury in db/db mice. Thus, our findings provided additional evidence for Smad3 as a pathogenic mediator and therapeutic target for T2D and T2DN.

In addition, we also identified a better inhibitory effect of AANG on NF-κB inflammatory pathway in the diabetic kidney due to its synergistic induction of Smad7 expression. It has been reported that AA can directly induce Smad7, and NG can also protect Smad7 from degradation by inhibiting Smad3-mediated Smurf2 mechanism 18. Because Smad7 is an inhibitor of NF-κB and overexpression of renal Smad7 can protect kidney to NF-κB -mediated renal inflammation under diabetic conditions 3, 7, 8, 23, thus, the synergistic effect of AANG on induction of Smad7 may be a mechanism through which AANG treatment produced a better inhibitor effect on renal inflammation in db/db mice. We have previously shown that Smad3 mediates renal fibrosis by altering a number of Smad3-dependent lncRNAs including upregulating a fibrogenic lnRNA Erbb4-IR and inflammatory LRNA9884 in db/db mice 19,20, 30. In the present study, we also revealed that inhibition of Erbb4-IR-mediated renal fibrosis and LRNA9884-dependent inflammation may be another mechanism by which preventive treatment with AAGN protected against T2DN.

It is possible that other signaling transducers such as the protein kinase B (Akt) and glycogen synthase kinase-3β (GSK-3β) pathways may be also the therapeutic targets of AANG. Indeed, a recent study identified that inhibition of GSK3β is likely able to improve islet beta cell homeostasis in diabetic mice 31. It has also been reported that treatment with AA or NG exerts its protective effects against ischemic or β-amyloid protein induced cell apoptosis via the Akt/GSK-3β/HIF-1α mechanisms 32-34. All these findings suggest that AA, NG, or AANG may work individually and additively to exert the inhibitor effects on T2D and T2DN via mechanisms involving multiple signaling pathways, which may require further investigation.

In conclusion, we identify a safe dosage and optimal combination ratio of AANG for preventive treatment of T2D and T2DN by effectively rebalancing Smad3/Smad7 signaling, thereby inhibiting TGF-β/Smad3-mediated renal fibrosis and NF-κB-driven renal inflammation. Unexpectedly, we also discover that treatment with AANG can protect islets from diabetic injury in db/db mice by promoting insulin-producing β cell development. Results from this study provide evidence and important clinical rationale for AANG as a novel and effective therapeutic strategy for diabetes and diabetic complications.

## Supplementary Material

Supplementary figures.

## Abbreviations

AA: Asiatic Acid
AGE: Advanced glycation end-products
ALT: Alanine aminotransferase
AST: Aspartate aminotransferase
Col-I: collagen-I
DN: Diabetic nephropathy
IPGTT: Glucose tolerance test
IPITT: insulin tolerance tests
LDH: Serum Lactate dehydrogenase
lncRNAs: long noncoding RNAs
mTEC: Mouse tubular epithelial cells
mMES: Mouse mesangial cells
MTT: methyl-thiazoldiphenyl tetrazolium
MCP-1: Monocyte Chemoattractant Protein-1
NG: Naringenin
PAS: Periodic Acid-Schiff
TCM: Traditional Chinese medicine
T2DN: Type-2 diabetic nephropathy
T2DN: Type-2 diabetes
UACR: Urine albumin-to-creatinine ratio
α-SMA: α-smooth muscle actin
