# Supplementary material for: AANG Prevents Smad3-dependent Diabetic Nephropathy by Restoring Pancreatic β-Cell Development in db/db Mice
Source: Int J Biol Sci. 2022 Aug 29;18(14):5489–502. doi: 10.7150/ijbs.72977 (PMC9461652; doi:10.7150/ijbs.72977)
Supplement: Supplementary file 1 — Supplementary figures. [file ijbsv18p5489s1.pdf]

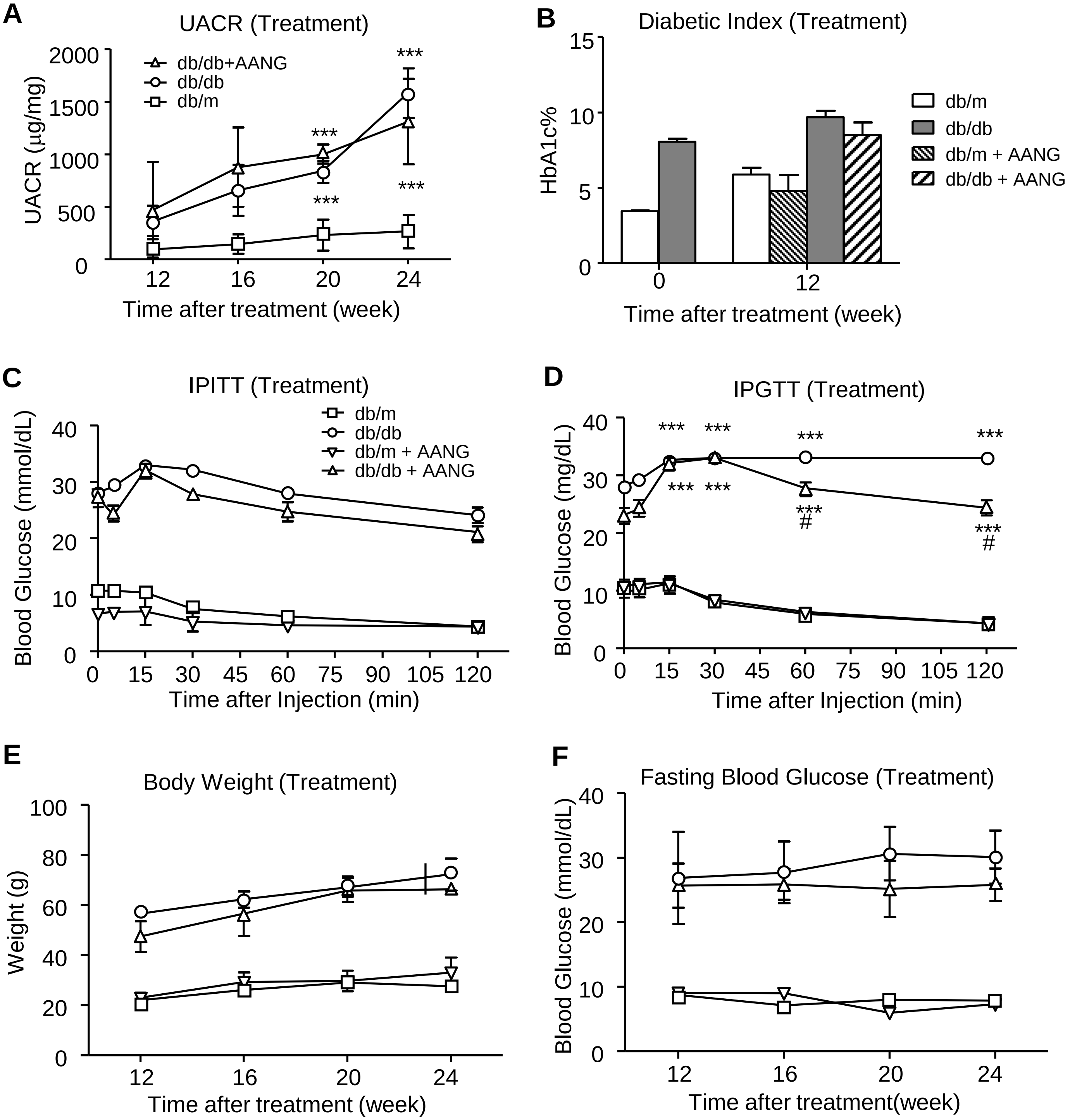

Supplementary Figure S1.Intervention therapy with AANG on established T2D shows no significant effect on T2D and T2DN in db/db mice. The 12-week-treatment with AANG from week 12-old db/db mice does improve: **(A)** UACR, **(B)** HbA1c, **(C)** insulin resistance (IPITT), **(E)** body weight, and **(F)** fasting blood glucose levels, although the glucose tolerance **(D)** is significantly improved by IPGTT assay. n=6. ***p < 0.001 vs control db/m mice; #p<0.05 vs control db/db mice.

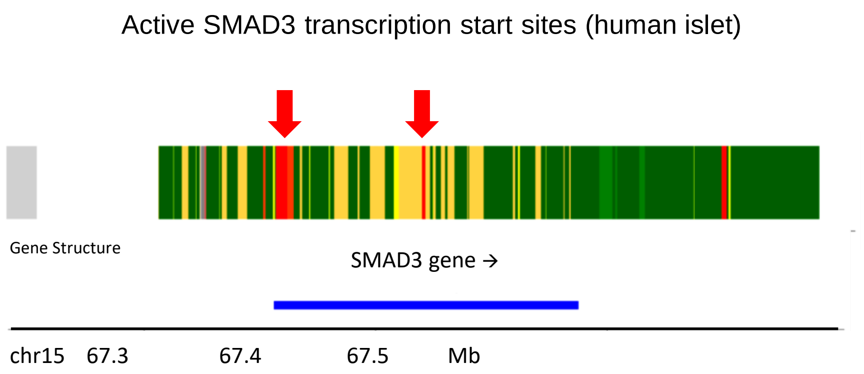

Supplementary Figure S2. Active transcription start sites are islet-specifically enriched at Smad3 genomic sequence in T2D patients (https://shinyapps.jax.org/endoc-islet-multi-omics/).

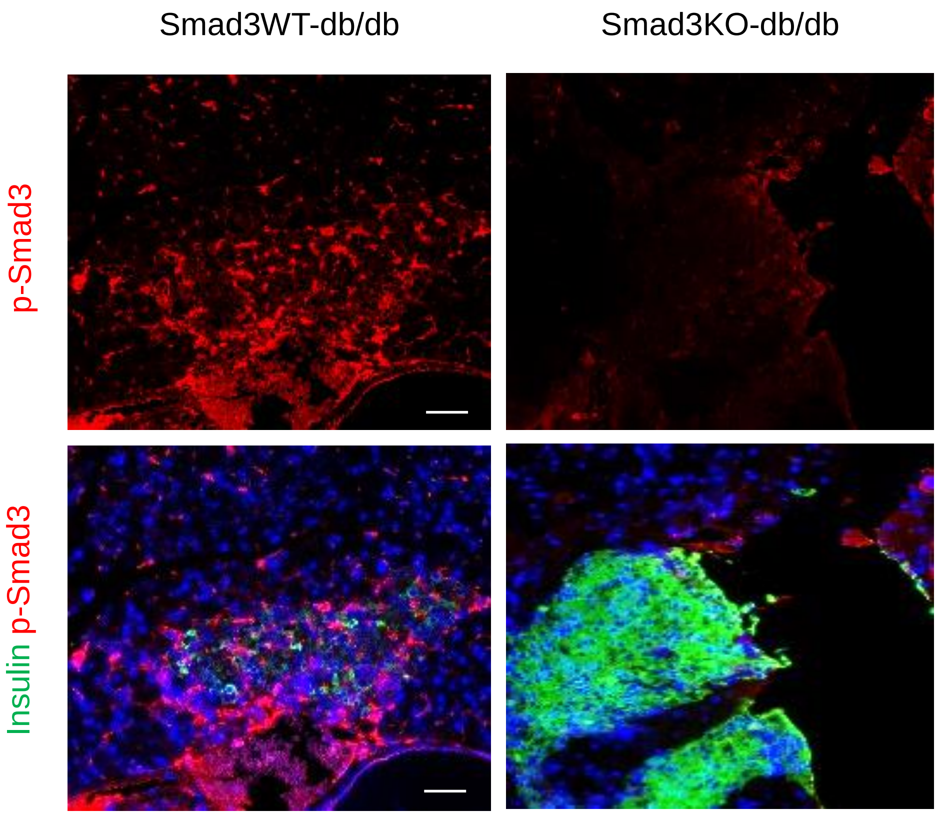

Supplementary Figure S3. Two-color immunofluorescence detects that in high contrast to the Smad3 WT-db/db mice, db/db mice lacking Smad3 (Smad3 KO-db/db) are protected islet β cells from diabetic injury by markedly increasing insulin-producing β cells (green). n=4. Scale bar, 50 µm.
